# ATAD5 deficiency alters DNA damage metabolism and sensitizes cells to PARP inhibition

**DOI:** 10.1093/nar/gkaa255

**Published:** 2020-04-16

**Authors:** Sara Giovannini, Marie-Christine Weller, Hana Hanzlíková, Tetsuya Shiota, Shunichi Takeda, Josef Jiricny

**Affiliations:** 1 Institute of Molecular Life Sciences of the University of Zurich, Winterthurerstrasse 190, 8057 Zurich, Switzerland; 2 Institute of Molecular Cancer Research of the University of Zurich, Winterthurerstrasse 190, 8057 Zurich, Switzerland; 3 Institute of Biochemistry of the Swiss Federal Institute of Technology, Otto-Stern-Weg 3, 8093 Zurich, Switzerland; 4 Department of Genome Dynamics, Institute of Molecular Genetics of the Czech Academy of Sciences, 142-20 Prague 4, Czech Republic; 5 Genome Damage and Stability Centre, School of Life Sciences, University of Sussex, Falmer, Brighton BN1 9RQ, UK; 6 Department of Radiation Genetics, Graduate School of Medicine, Kyoto University, 606-8501 Kyoto, Japan

## Abstract

Replication factor C (RFC), a heteropentamer of RFC1-5, loads PCNA onto DNA during replication and repair. Once DNA synthesis has ceased, PCNA must be unloaded. Recent findings assign the uloader role primarily to an RFC-like (RLC) complex, in which the largest RFC subunit, RFC1, has been replaced with ATAD5 (ELG1 in *Saccharomyces cerevisiae*). ATAD5-RLC appears to be indispensable, given that *Atad5* knock-out leads to embryonic lethality. In order to learn how the retention of PCNA on DNA might interfere with normal DNA metabolism, we studied the response of ATAD5-depleted cells to several genotoxic agents. We show that ATAD5 deficiency leads to hypersensitivity to methyl methanesulphonate (MMS), camptothecin (CPT) and mitomycin C (MMC), agents that hinder the progression of replication forks. We further show that ATAD5-depleted cells are sensitive to poly(ADP)ribose polymerase (PARP) inhibitors and that the processing of spontaneous oxidative DNA damage contributes towards this sensitivity. We posit that PCNA molecules trapped on DNA interfere with the correct metabolism of arrested replication forks, phenotype reminiscent of defective homologous recombination (HR). As *Atad5* heterozygous mice are cancer-prone and as *ATAD5* mutations have been identified in breast and endometrial cancers, our finding may open a path towards the therapy of these tumours.

## INTRODUCTION

*ATAD5* is the human homolog of the *Saccharomyces cerevisiae* gene *ELG1* (Enhanced Level of Genomic instability), which was first identified as a suppressor of direct repeat recombination (1). In later studies, its loss was shown to be synthetically lethal in genome-wide screens carried out with *mus81* or *mms4* deletion mutants (2), or in a candidate screen designed to identify genes that suppress gross chromosomal rearrangements (GCRs) (2,3). *ELG1* defect was thus associated with hyper-recombination. Because the Mus81/Mms4 heterodimer has been implicated in the processing of branched DNA structures such as those arising during the rescue of stalled replication forks (4) and because GCRs are believed to result from erroneous processing of double-strand DNA breaks (DSBs) caused by replication fork collapse (5), Elg1 was predicted to play a protective role at the replication fork and this prediction was substantiated in subsequent studies.

At the onset of DNA replication, RFC1-5 loads the homotrimeric proliferating cell nuclear antigen (PCNA) sliding clamp, the processivity factor of DNA polymerases δ and ϵ, onto DNA (6,7). PCNA is also required in the gap-filling steps of mismatch repair, nucleotide excision repair or long-patch base excision repair, as well as during recombination (8). Upon completion of DNA synthesis, PCNA must be unloaded and it has long been believed that this function is fulfilled by RFC. Indeed, RFC1-5, RFC2-5 and even RFC2,5 have all been reported to unload PCNA from double-stranded DNA *in vitro* (9,10) [reviewed in (7)]. However, experimental evidence obtained initially in *S. cerevisiae* suggested that PCNA unloading *in vivo* is catalysed primarily by a complex of Rfc2-5 and Elg1 (11).

RFC1, the largest RFC subunit, has three orthologs: Rad24 (RAD17 in human), Ctf18 and Elg1, all of which can interact with the RFC2-5 subunits to form RFC-like complexes, RLCs (2,3,12), which are functionally-redundant in activating the S phase checkpoint in response to stress induced by hydroxyurea (HU) or methyl methanesulphonate (MMS) (3). Rad24-RLC has been shown to load onto DNA the Ddc1/Rad17/Mec3 alternative sliding clamp (13), while Elg1-RLC and Ctf18-RLC have been assigned roles in chromatid cohesion (14,15). The Elg1-RLC complex interacts directly with PCNA (3) and because Δ*elg1* strains accumulate PCNA in chromatin and Elg1-RLC can unload it (11), the latter complex has been assigned the role of PCNA unloader. Moreover, it appears to display a preference for post-translationally-modified (ubiquitylated or SUMOylated) PCNA (16,17). Because these modifications accompany replication fork stalling and facilitate lesion bypass (18), it is possible that, while unmodified PCNA is unloaded by RFC, its modified isoforms may be removed by Elg1-RLC. In the absence of this complex, the sliding clamp remains on chromatin beyond S phase (19) and interferes with normal DNA metabolism, which was suggested to lead to the observed genomic aberrations [reviewed in (20,21)].

Studies analyzing ATAD5 deficiency in mammalian systems yielded findings similar to those described for its yeast homolog Elg1. ATAD5 siRNA-depleted cells accumulated spontaneous DNA damage and displayed a delay in S phase, whereby their replication factories were shown to persist into the G2 phase. ATAD5 was reported to be stabilised upon exposure of cells to UV, aphidicolin, HU and MMS, and to form foci that co-localized with stalled replication forks detected by BrdU labeling (22). The depleted cells accumulated PCNA and ubiquitylated PCNA in chromatin, and as ATAD5-RLC was shown to physically interact with the Usp1 deubiquitylase, it was suggested that the protein complex played a role in controlling DNA damage bypass, which requires mono-ubiquitylated PCNA (23). Some support for this role was obtained recently by showing that ATAD5-RLC is able to unload both unmodified and modified PCNA isoforms from DNA (24).

Puzzlingly, the ATAD5 siRNA-depleted cells were shown to display higher levels of spontaneous homologous recombination (HR), but reduced levels of DSB-induced HR in reporter assays using *I-Sce* nuclease, which suggested that the protein complex has different roles in the metabolism of intermediates generated during these two processes (22). More recently, ATAD5-depleted cells were reported not to form RAD51 foci following exposure to ionizing radiation (25), which was interpreted as a sign of defective HR.

We set out to study the phenotype of *ATAD5*-deficient cells in an attempt to understand how ATAD5-RLC, a protein complex that is not a known member of the recombination machinery, could affect the outcome of HR events. We were also interested in identifying the nature of the spontaneous events that cause genomic instability in ATAD5-deficient cells. Moreover, given that *Atad5* heterozygous mice are cancer-prone and that *ATAD5* mutations have been detected in tumors of the breast, ovary (26) and endometrium (27), we wondered whether ATAD5 deficiency could be exploited therapeutically. Here, we show that ATAD5-depleted cells are hypersensitive to the interstrand cross-linking agent mitomycin C (MMC) and to inhibitors of poly(ADP)ribose polymerases (PARPs), which are successfully used in the therapy of tumors deficient in HR. We also show that the metabolism of oxidative base damage contributes towards the toxicity of PARP inhibitors in ATAD5-depleted cells.

## MATERIALS AND METHODS

### Cell culture and treatments

A2780 cells stably-expressing the tetracycline repressor (a generous gift of Lynne Hu and Milica Enoiu) were transfected either with an expression plasmid containing shRNA targeting the ATAD5 sequence in exon 2 (^5′^CUGACGAUGUACAAGAUAAUA^3′^), or a similar vector containing an shRNA targeting ATAD5 sequence in the 3′UTR (^5′^GUAUAUUUCUCGAUGUACA^3′^) under the control of a tetracycline-inducible promoter. One stable clone (1–6) from the former transfection and one (15-3) from the latter were expanded and used in the experiments described below. The cells were grown in DMEM (Gibco) supplemented with 5% Tet-off fetal calf serum (FCS, Sigma), penicillin (100 U/ml, Gibco) and streptomycin (100 μg/ml, Gibco), and selected with blasticidin (10 mg/ml, InvivoGen) and puromycin (10 mg/ml InvivoGen) at 37°C in a 5% CO_2_ humidified atmosphere. For induction of ATAD5 knock-down, cells were exposed to 50 ng/ml doxycycline (Dox, Clontech) for at least 4 days.

U2OS cells were grown in DMEM (Gibco) supplemented with 10% FCS, streptomycin and penicillin (each 100 U/ml).

The DT40 *ATAD5^−^^/^^−^* cells were created by replacing exons 9–16 encoding 896–1286 amino acids of the chicken *ATAD5* gene by selection marker genes (Supplementary Figure S2C). The desired gene disruption was verified by Southern blot analysis (Supplementary Figure S2D) and RT-PCR (Supplementary Figure S2E) using the primers Atad5-sense: ^5′^AAAATCGCCATCTCACTTGG^3′^ and Atad5-antisense: ^5′^CACAGCCTGAGTCACATTTTGG^3′^. The cells were cultured in RPMI (Gibco) supplemented with 10% FCS, streptomycin and penicillin (each 100 U/ml), chicken serum and β-mercaptoethanol as described previously (28).

MMS was used at the indicated concentrations during the clonogenic assay, and 0.01% for 1 h before protein detection by Western blots. Bleomycin sulphate (10 mg, Merck CAS 9041-93-4) was dissolved in 1 ml PBS and stored in −20°C. After dilution in DMEM, it was added to cells at the indicated final concentrations and removed after 1 h. CPT (10 mM stock solution) was used at the concentrations indicated in the clonogenic assays. It was not washed out after the treatment and the cell culture medium was not replaced during the duration of the assay. For RPA and 53BP1 foci detection, CPT concentration was 1 μM and the treatment lasted 1 h. For MMC treatment, cells were exposed to the indicated concentrations for 48 h. They were then washed with PBS and cultured in fresh medium for the duration of the experiment. Olaparib (AZD2281, Ku-0059436; or S1060, Selleckchem, 20 mM stock solution in DMSO) was stored at −80°C. For the treatments, it was diluted in DMEM and added to cells at the indicated final concentrations. Veliparib (10 mM stock solution in DMSO) and PJ34 (10 mM stock solution in sterile ddH_2_O) were stored at −80°C. For cell treatments, the PARPis were diluted in DMEM and added to cells at the indicated final concentrations.

The reactive oxygen species (ROS) scavenger *N*-acetylcysteine (NAC, Sigma) was added to the cell culture medium at a final concentration of 2.5 mM. Cells were treated first for 24 h and then again for 30 min before the comet assay was performed or before olaparib addition for the clonogenic assay.

### siRNA transfections

Cells were seeded to approximately 30–50% confluency and transfected with 40 pmol siRNA oligonucleotides using Lipofectamine RNAiMAX™ (Invitrogen) according to the manufacturer's instructions. The following oligonucleotides were used:

siLuciferase (siLuc): ^5′^CGUACGCGGAAUACUUCGA^3′^;
siATAD5: ^5′^GUAUAUUUCUCGAUGUACA^3′^;
siPARP1 (29): ^5′^AAGCCAUGGUGGAGUAUGA^3′^;
siMYH: ^5′^UCACAUCAAGCUGACAUAUCAAGUA^3′^;
siOGG1: ^5′^UCCAAGGUGUGCGACUGCUGCGACA^3′^ (all from Microsynth, Balgach, Switzerland).

### Clonogenic assays

A2780 and U2OS cells were seeded in triplicates in 6-well plates at a density of 300–500 cells per well 72 h after siRNA transfection. After adhesion, cells were treated as described above with the indicated concentrations of each drug and incubated at 37°C. Colony growth was stopped after 10–14 days. Cells were washed with PBS and incubated with 0.5% Crystal violet in 20% EtOH for 15 min at room temperature (RT). Crystal violet was removed and gently washed away with water. Colonies were counted when dry.

DT40 cells were seeded in methylcellulose pre-mixed with the indicated drug concentrations. Colonies were counted 10–14 days after seeding.

Cell survival of treated cells is reported as percentage of untreated cells in a line chart, showing an average of three independent assays with standard deviation and significance (*P < 0.05; **P < 0.01; ***P < 0.001; ****P < 0.0001) calculated by two-tailed Student's *t* test.

### Alkaline comet assays

The CometAssay^®^ kit from Trevigen^®^ was used according to the manufacturer's instructions. 72 h after transfection with siRNA, the cells were re-suspended in ice-cold PBS at a concentration of 3 × 10^5^ cells/ml, embedded in molten LM agarose at a ratio of 1:10 and spread on CometSlides™. The slides were immersed in 4°C Lysis Solution overnight before exposure to Alkaline Unwinding Solution (300 mM NaOH, 1 mM EDTA, pH > 13) for 1 h at 4°C. Electrophoresis at 21 V for 30 min was performed in the specific Electrophoresis buffer. The slides were washed twice in dH_2_O and immersed in 70% EtOH for 5 min after electrophoresis. All slides were finally dried at 37°C and stained with SYBR^®^ Green for 30 min. Images were captured with an Olympus IX81 fluorescence microscope and at least 100 cells per condition were analysed in each of three independent experiments by ImageJ software. The average of three independent assays with standard deviation and significance (*P < 0.05; **P < 0.01; ***P < 0.001; ****P < 0.0001) calculated by two-tailed Student's *t* test is reported in a bar chart.

### Protein extractions and western blots

Cells seeded in 10 cm dishes and treated as indicated were harvested, the pellet was washed in cold PBS and quickly frozen in liquid nitrogen. Chromatin extracts were prepared with cold pre-extraction buffer (25 mM HEPES, pH 7.4, 50 mM NaCl, 1 mM EDTA, 3 mM MgCl_2_, 300 mM sucrose, 0.5% Triton-X-100) supplemented with protease and phosphatase inhibitors. After centrifugation at 10 000 × g, the cytoplasmic fraction was removed and the pellets were re-suspended in cold SDS lysis buffer (1% SDS, 10 mM Tris–HCl, pH 8) with protease and phosphatase inhibitors, and finally sonicated (20 s, 50 cycles, 70% amplitude, Bandelin Sonoplus).

Whole cell extracts were prepared by re-suspending the cell pellets in SDS lysis buffer prior to sonication and centrifugation at 18 000 × g to clear the lysate. Protein concentration was determined by the Lowry assay: samples were diluted in dH_2_O and incubated for 10 min with a mixture (50:1) of Solution A (2% Na_2_CO_3_ in 0.1 N NaOH) and B (0.5% CuSO_4_^.^5H_2_O in 1% sodium citrate). 100 μl Folin & Ciocalteau's phenol reagent (Sigma) diluted 1:2 with H_2_O was added and the mixture was incubated for 30 min at RT. Absorbance was measured at 750 nm (Varian-Cary 50 Scan spectrophotometer) and protein concentration was calculated from a BSA standard curve.

After 5 min incubation at 95°C with Loading Buffer (5×, 0.25 M Tris pH 6.8, 50% glycerol, 8% SDS, 0.5 mM DTT, 0.1% bromophenol blue), 25–30 μg of total protein were separated according to size on polyacrylamide gels (6% for ATAD5) or on precast 4–12% gels (BioRad) using the Mini Trans-Blot Electrophoretic Transfer Cell (BioRad) in 10% SDS-buffer at 60 V. Proteins were transferred in Transfer Buffer (25 mM Tris, 192 mM glycine, 20% MeOH) overnight at 30 V at 4°C onto a Hybond-P Polyvinylidene fluoride (PVDF) membrane (Amersham Pharmacia Biotech) pre-activated in 100% MeOH. Membranes were blocked in 5% non-fat dry milk or BSA (depending on the antibody) in TBS-T (20 mM Tris–HCl pH 7.4, 150 mM NaCl and 0.1% Tween-20) for 30 min at RT before incubation with primary antibodies overnight at 4°C. The membranes were washed three times with TBS-T and subsequently incubated with secondary antibodies (HRP-conjugated sheep anti-mouse or donkey anti-rabbit IgG, GE Healthcare) for 1 h at RT. After three washes in TBS-T, the membranes were incubated for 1 min with WesternBright™ Chemiluminescent Detection Reagent (Advansta) and analysed by Fusion Solo (Vilber Lourmat) imager.

The antibodies used were: ATAD5 (Rabbit, dilution 1:300, a kind gift of Maite Oliveira-Harris); Lamin B1 (Rabbit, dilution 1:1000, Abcam ab16048); PARP1 (Rabbit, dilution 1:1000, Santa Cruz, sc7150); PCNA (Mouse Santa Cruz sc56). HRP-conjugated secondary anti-mouse and anti-rabbit antibodies (GE Healthcare) were used at a dilution of 1:5000.

### Immunoprecipitation

Cells pellets were incubated on ice for 15 min in pre-extraction buffer (25 mM HEPES, pH 7.4, 50 mM NaCl, 1 mM EDTA, 3 mM MgCl_2_, 300 mM sucrose, 0.5% Triton-X-100), supplemented with protease and phosphatase inhibitors. After centrifugation (5000 × g, 5 min) and removal of the supernatant, chromatin pellets were resuspended in RIPA buffer (10 mM Tris–HCl pH7.5, 150 mM NaCl, 5 mM EDTA, 0.1% SDS, 1% Triton-X-100, 1% sodium deoxycholate) supplemented with protease and phosphatase inhibitors. The lysates were homogenized and incubated with Benzonase for 1 h on a rotator at 4°C. After centrifugation (18 000 × g, 10 min), the supernatant was collected and used for protein concentration measurement. 300 μg of the extract were resuspended in 200 μl RIPA buffer and 100 μl dilution/washing buffer (10 mM Tris–HCl pH7.5, 150 mM NaCl, 5 mM EDTA) supplemented with protease and phosphatase inhibitors. Samples were then incubated with 2 μg of anti-PCNA antibody (Santa Cruz, sc56) on a rotator overnight at 4°C.

Protein G agarose beads were washed three times for 5 min with washing buffer and incubated with the samples for 3 h on a rotator at 4°C. After three washes, proteins were eluted with loading buffer and incubated for 5 min at 95°C on a thermomixer. After 1 min centrifugation at 18 000 × g, the supernatant was transferred into a new tube, and the proteins were loaded onto a gel for detection by electrophoresis.

### Immunofluorescence

Cells were plated on sterilized coverslips and treated the next day with either DMSO as a vehicle or 10 μM poly(ADP)ribose glycohydrolase (PARG) inhibitor PDD 0017273 (Tocris Bioscience, DMSO solution) for 30 min. After washing with PBS, the cells were fixed with 4% formaldehyde in PBS for 10 min at RT. A subsequent permeabilization was performed in ice-cold methanol/acetone solution (1:1) for 5 min prior to PBS washing and 1 h blocking in 10% FBS. Coverslips were then incubated for 1 h with the primary antibodies (anti-PAR sc7150, Trevigen 4336-BPC-100 rabbit, diluted 1:1000; anti-PCNA sc56, mouse, diluted 1:1000) in 10% FBS/PBS. After PBS washing, incubation with secondary antibodies (AlexaFluor 488, A11008, goat anti-rabbit, diluted 1:1100; AlexaFluor 568, A10037, donkey anti-mouse, diluted 1:1000) lasted 1 h in the dark. Coverslips were washed (3 × 5 min in PBS), stained with DAPI and mounted using VECTASHIELD (Vector Laboratories). Automated wide-field image acquisition was done using Olympus ScanR high-content screening station equipped with a motorized stage and 40× objective. Nuclei were identified based on the DAPI signal and PCNA-positive cells were gated and quantified using ScanR Analysis Software. At least 100 nuclei were counted per condition in three independent experiments. Data are represented as mean ± SEM.

To visualise DNA damage foci, the cells were plated in 6-well plates on sterilized coverslips and treated the day after with 1μM CPT for 1 h. After washing with cold PBS, pre-extraction/fixation were performed in 100% ice-cold MeOH for 15 min. Permeabilization was done in 0.3% Triton/PBS for 10 min prior to washing with PBS and 30 min blocking in 3% BSA/PBS. Coverslips were then incubated for 2 h with the primary antibodies (Anti-RPA antibody, Abcam ab79398, rabbit, diluted 1:1000; Anti-53BP1 antibody, Abcam ab36823, rabbit, diluted 1:1000) in 3% BSA/PBS in a humid chamber. After washing with cold PBS, incubation with secondary antibodies (AlexaFluor 568, A11036, goat anti-rabbit, diluted 1:100; AlexaFluor 488, A11029, goat anti-mouse, diluted 1:100) lasted 1 h in the dark in a humid chamber. Mounting medium with DAPI (VECTASHIELD, Vector Laboratories) was added after the last washing step. Images were acquired using a wide-field Leica DM6B microscope (HCX PL APO 63× objective). At least 100 cells per condition were analysed. The average of three independent assays with standard deviation and significance (*P < 0.05; **P < 0.01; ***P < 0.001; ****P < 0.0001) calculated by two-tailed Student's *t* test are shown.

### Proliferation assay

10^5^ cells were seeded in 10 cm dishes and harvested for counting at the indicated times. Cell proliferation was then shown as fold increase relative to the initial cell number at 0 h time point.

### Cell cycle analysis by flow-cytometry (PI)

After cell treatment with Dox and/or olaparib, the cells were harvested and counted. 2 × 10^6^ cells were then washed with cold PBS, resuspended in 80% cold EtOH and stored at 4°C. After centrifugation, the cells were incubated at 37°C with 500 μl of PI solution in PBS (50 μg/ml Propidium Iodide, 0.1 mg/ml RNaseA, 0.05% Triton-X-100, PBS) for 30 min in the dark. After addition of PBS, centrifugation and supernatant removal, the cells were resuspended in 500 μl PBS and transferred to round-bottom tubes for subsequent flow-cytometric analysis.

## RESULTS AND DISCUSSION

### ATAD5 deficiency sensitizes cells to a range of genotoxic chemicals

We wanted to learn whether ATAD5-deficiency sensitized cells to clinically-relevant DNA damaging agents. However, *Δelg1* yeast cells (1–3) and mammalian cells lacking ATAD5 (27) were reported to display genomic instability. Thus, rather than generate a human knock-out cell line that might acquire deleterious genetic defects over a period of time in cell culture, we decided to establish a stable cell line, in which ATAD5 deficiency could be induced when required. To this end, we made use of an ovarian carcinoma cell line A2780 (ECACC catalogue no. 93112519), which is frequently used in drug sensitivity tests. We used a clone that stably-expresses the tetracycline repressor (a generous gift of Lynne Hu and Milica Enoiu) and transfected it with vectors carrying two different ATAD5 shRNAs under the control of doxycycline (Dox)-inducible promoter (see Materials and Methods). Two stable clones, 1–6 and 15–3, were isolated (one from each transfection) and their growth characteristics were found to be similar to those of the parent A2780 clone (data not shown), but slowed down substantially when ATAD5 was depleted (Supplementary Figure S1A), as reported also by others (30).

**Figure 1.**
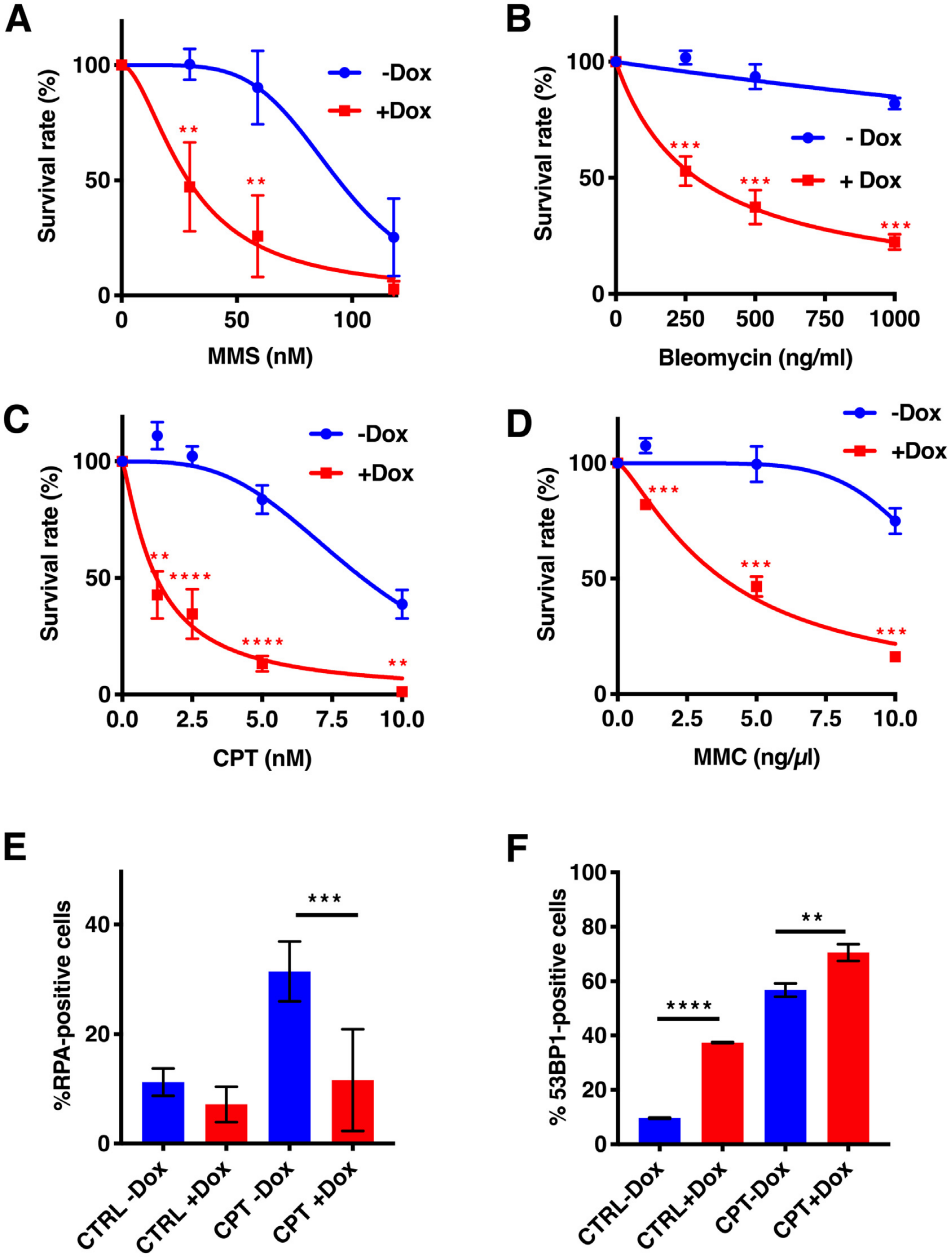
Differential effect of ATAD5 depletion on chemical sensitivity of A2780 cells. Knock-down of ATAD5 in human ovarian carcinoma cell line A2780 clone 1–6 was achieved by inducing the expression of ATAD5 shRNA with doxycycline (+Dox). DMSO (–Dox) was used as a control (see Supplementary Figure S1B). Four days later, the cells were exposed to the respective chemical and the colonies were counted 10–14 days later (see Materials and Methods). The figure shows the results of clonogenic assays after treatment with MMS (**A**), bleomycin (**B**), CPT (**C**) and MMC (**D**). The values represent percentages of drug-treated versus untreated colonies in the Dox-uninduced or Dox-induced cell populations, respectively. Panels **E** and **F** show the percentages of cells displaying RPA- or 53BP1 foci, respectively, in untreated and CPT-treated cell populations (see also Supplementary Figure S1 D,E). The data represent means of at least three independent experiments, each carried out in triplicate ± SD. Asterisks indicate levels of statistical significance calculated by two-tailed Student's *t* test (*P*-value < 0.05 *, < 0.01 **, < 0.001 ***, <0.0001 ****).

Treatment of clone 1–6 with Dox caused a substantial downregulation of ATAD5 expression within four days as measured by Western blots (Supplementary Figure S1B). Like cells depleted of ATAD5 by siRNA (22), the Dox-treated A2780 clone 1–6 was sensitized to MMS (Figure 1A and Supplementary Figure S1C), which helped confirm the validity of our experimental system. Given that MMS has often been referred to as a radiomimetic (31), the latter result would appear to imply that ATAD5-deficient cells are unable to efficiently repair double strand breaks (DSBs) generated by this chemical. We wanted to verify this finding by testing the response of our cells to a *bona fide* radiomimetic, bleomycin. As shown in Figure 1B, the ATAD5-deficient cells were indeed hypersensitive to this agent.

Both the above reagents generate single-strand breaks (SSBs) in DNA. MMS methylates purines (32), which are subsequently either spontaneously lost or removed by 3-methyladenine DNA glycosylase. The resulting abasic sites are then converted to SSBs by AP-endonuclease (33,34). Bleomycin generates SSBs by cleaving the sugar moieties of the DNA backbone *via* a free radical mechanism involving the reduction of two co-ordinated iron atoms. The collision of these SSBs with the replication fork would give rise to one-ended DSBs, which would be addressed during S phase by homologous recombination (HR) (35). In contrast, should two SSBs arise in close proximity on opposite strands, they would give rise to DSBs that would be addressed primarily by the non-homologous end-joining (NHEJ) repair pathway. Interestingly, while CFP-ATAD5 was seen to localize to arrested replication forks shortly after treatment with low dose MMS, the γ-H2AX foci (markers of DSBs) induced by this reagent at later time points post-treatment did not colocalize with the CFP-ATAD5 signal (22). This suggested that the cytotoxicity of these two substances in cells lacking ATAD5-RLC was due to the defective processing of arrested replication forks, rather than by the misrepair of DSBs arising at sites distal to the replication forks.

The ATAD5-depleted cells were also hypersensitive to CPT (Figure 1C) and MMC (Figure 1D). CPT is a topoisomerase I inhibitor that causes toxicity in S phase through collisions between unligated topoisomerase-induced SSBs and replication forks (36), while MMC is a cancer therapeutic that causes toxicity by generating interstrand DNA cross-links (ICLs) (37). The latter lesions are addressed predominantly by the *Fanconi anaemia* (FA) pathway, which is activated upon the collision between ICLs and replication forks (38). Taken together, our results suggest that ATAD5-RLC affects the efficiency of repair of DSBs arising at the replication fork and is consistent with the recent identification of ATAD5 in the proteome associated with nascent DNA (39).

The above evidence suggested that ATAD5-deficient cells might have a defect in HR. In an attempt to substantiate this prediction, we examined DNA damage foci generated by CPT in ATAD5-expressing and ATAD5-depleted cells. Intense RPA foci were readily detectable in ∼10% ATAD5-expressing cells and this percentage increased to ∼35% upon CPT treatment. In contrast, ATAD5-depleted cells displayed slightly fewer RPA foci than control cells and although this number increased upon CPT treatment, the focus intensity did not (Figure 1E, and Supplementary Figure S1D). The decreased intensity of the foci is likely indicative of reduced resection during HR. In contrast, both the number and intensity of foci of 53BP1 indicative of NHEJ increased upon ATAD5 knock-down in CPT-treated cells (Figure 1F and Supplementary Figure S1E). Taken together, this evidence suggests that ATAD5-RLC deficiency and the concomitant accumulation of PCNA in chromatin interferes with the DSB resection or replication fork remodelling processes, both of which give rise to long stretches of ssDNA that are bound by RPA. This would hinder HR and the cells would be increasingly dependent on NHEJ for DSB repair, as seen in the increase in the number of 53BP1 foci. This forced switch in DSB repair pathway choice may help explain the phenotype of Elg1/ATAD5-depleted cells, which have been reported to be hyper-recombinogenic and display extensive genomic instability [reviewed in (21,40)], but also to have abnormal HR in reporter assays (22).

### ATAD5-deficient cells are hypersensitive to PARP inhibitors

HR-deficient cells are highly-sensitive to inhibitors of poly(ADP)ribose polymerases (PARPis) (41,42), which represent a new class of compounds used in the therapy of ovarian and breast tumours carrying mutations in the *BRCA* genes (43). PARPs potentiate the repair of SSBs arising during base excision repair (BER) (44), and mediate Okazaki Fragment (OF) ligation (45). Unrepaired breaks and nicks colliding with replication forks give rise to one-ended DSBs, the toxicity of which is normally rescued with the help of HR, but, in cells lacking HR, PARPs inhibition leads to accumulation of unrepaired or misrepaired DSBs and cell death.

**Figure 2.**
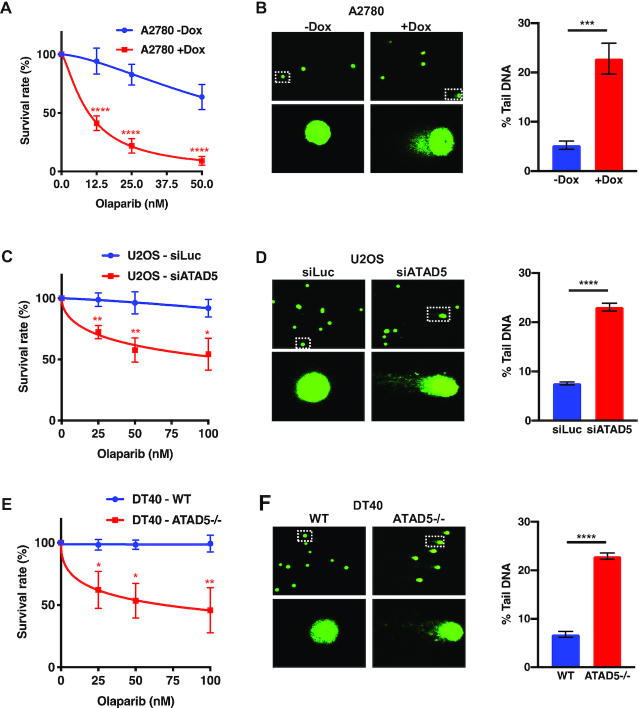
ATAD5-depleted or -deficient cells are hypersensitive to the PARP inhibitor olaparib and display genomic instability. Sensitivity and genomic instability of ATAD5-depleted cells were estimated by clonogenic (panels **A**, **C** and **E**) and alkaline comet (panels **B**, **D** and **E**) assays (see Materials and Methods). (A, B) Human ovarian carcinoma cell line A2780 clone 1–6 +/− Dox; (C, D) Human osteosarcoma U2OS cells pre-treated with siRNA against luciferase (siLuc, control) or ATAD5 (siATAD5); (E, F) DT40 cells, wild type (WT) or *ATAD5^−^^/^^−^*. The values represent percentages of olaparib-treated versus untreated colonies in the Dox-uninduced or Dox-induced cell populations, respectively. The data represent means of at least three independent experiments, each carried out in triplicate ± SD. Asterisks indicate levels of statistical significance calculated by two-tailed Student's *t* test (*P*-value < 0.05 *, < 0.01 **, < 0.001 ***, <0.0001 ****).

Given the apparent HR defect in our ATAD5-depleted cells, we decided to test whether they are sensitive to PARPis like *bona fide* HR-deficient cells. The ATAD5-depleted A2780 cells were clearly hypersensitive to the PARPi olaparib in clonogenic assays (Figure 2A and Supplementary Figure S2A) and displayed an increase in strand discontinuities as measured by an alkaline comet assay (Figure 2B). In order to verify that the observed phenotype was not restricted solely to this cell line, we repeated the experiment with U2OS (human osteosarcoma) cells, in which ATAD5 was knocked-down with siRNA (Supplementary Figure S2B). The outcome was similar in both clonogenic (Figure 2C) and comet assays (Figure 2D). In parallel, we knocked-out the *ATAD5* gene in chicken DT40 cells (Supplementary Figure S2C–E). Like cells from other species (19,30), the *ATAD5^−^^/^^−^* DT40 cells accumulated PCNA in their chromatin (Supplementary Figure S2F) and displayed hypersensitivity to olaparib (Figure 2E) and higher levels of genomic instability in comet assays (Figure 2F).

### Olaparib toxicity to ATAD5-depleted cells is caused by trapping of PARP1 on chromatin

**Figure 3.**
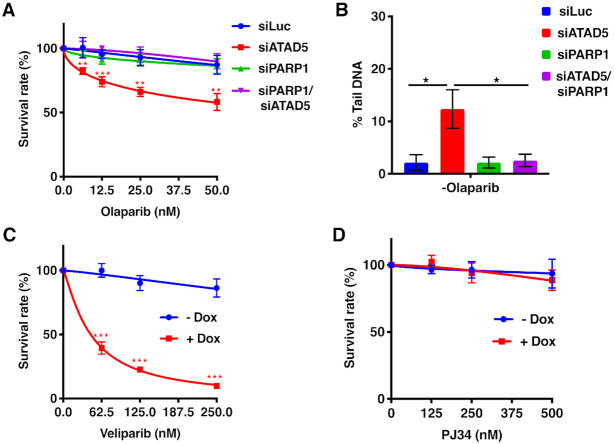
The synthetic lethality between PARP inhibition and ATAD5 deficiency is is attenuated by PARP depletion in U2OS cells and is mediated by PARP trapping on DNA. (**A**) U2OS cells treated with siRNA against ATAD5 were sensitive to olaparib (red line). Cells in which PARP1 (green line) or both ATAD5 and PARP1 (purple line) were knocked-down with siRNA were not more sensitive to olaparib than control cells treated with siLuc (blue line). (**B**) Alkaline comet assay showing the extent of genomic instability in the U2OS cells depleted of the indicated proteins. (**C**, **D**) Results of clonogenic assays showing the sensitivity of the ATAD5-depleted A2780 cells to Veliparib (C) and PJ34 (D). The values in panels A, C and D represent percentages of PARPi-treated versus untreated colonies in the Dox-uninduced or Dox-induced cell populations, respectively. The data represent a mean of at least three independent experiments, each carried out in triplicate ± SD. Asterisks indicate levels of statistical significance calculated by two-tailed Student's *t* test (*P*-value < 0.05 *, < 0.01 **, < 0.001 ***).

The toxicity of PARPis has been ascribed to both the catalytic inhibition of PARPs and the trapping of the inhibited PARP on DNA, where it interferes with DNA metabolism (46,47). Indeed, chromatin extracts of the ATAD5-depleted A2780 cells treated with olaparib contained higher amounts not only of PCNA, but also of PARP1, as shown by immunoprecipitation analysis (Supplementary Figure S3A, lane 4, cf. lane 2). In order to confirm that the toxicity of olaparib seen in ATAD5-deficient cells was related to inhibition of PARP1 and its trapping on DNA, we decided to knock-down PARP1 with siRNA. This would be predicted to reduce the toxicity of the drug. We used the human U2OS cells in this experiment, due to their superior transfection efficiency. The siRNA-mediated ATAD5 knock-down was efficient (Supplementary Figure S2B) and was accompanied by accumulation of PCNA in the chromatin of the ATAD5-depleted cells (Figure 3A). Treatment with siRNA against PARP1 was also successful, both when used alone and in combination with ATAD5 siRNA (Figure 3B). Clonogenic assays revealed that olaparib toxicity seen in ATAD5-depleted cells was rescued by PARP1 depletions (Figure 3C), as was genomic instability measured by alkaline comet assays (Figure 3D). We further confirmed these findings by studying the response of the A2780 clone 1–6 to two other PARPis, veliparib, which binds DNA weaker than olaprib, and PJ34, which does not bind DNA. Clonogenic assays showed that the ATAD5-depleted cells were sensitive to veliparib, although at around 5-times higher concentration compared to olaparib (Figure 3C). No sensitivity to PJ34 was detected (Figure 3D). Taken together, these data confirm that the toxicity of PARPis to ATAD5-deficient cells is mediated by trapped PARP complexes on DNA, as in the case of other HR-deficient cells (46).

### ATAD5 deficiency does not affect maturation of Okazaki Fragments

The Elg1/ATAD5-RLC has been assigned a number of roles, most of them associated with replication. It was suggested to help unload PCNA after OF ligation (48), or ubiquitylated PCNA after bypass of blocked forks (23), and to participate in the repair of DSBs both DNA damage-induced and spontaneous (22). Elg1-RLC has also been shown to participate in chromatin remodeling (49). It was postulated that these functions might be responsible for the slower progression of the ATAD5-depleted cells through S phase, even though there appeared to be no detectable effect on the speed of replication forks (30).

**Figure 4.**
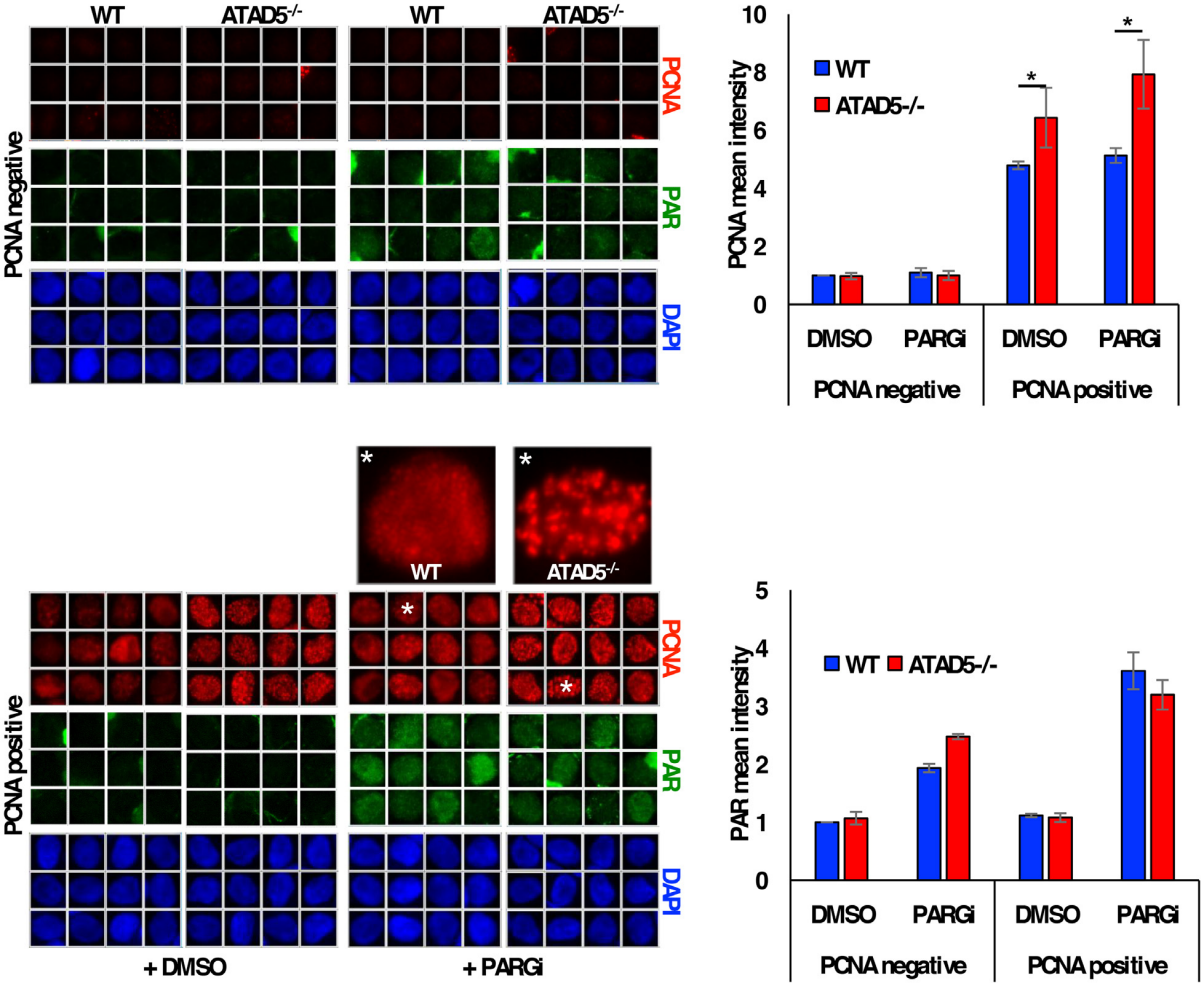
ATAD5 deficiency gives rise to prominent nuclear PCNA S phase foci in DT40 *ATAD5^−^^/^^−^* cells, but does not further activate PARPs during Okazaki Fragment maturation. Indirect immunofluorescence analysis of DT40 wild type or *ATAD5^−^^/^*^−^ cells treated with the poly(ADP)ribose glycohydrolase inhibitor (PARGi) or with DMSO (control). PCNA staining shows an accumulation of large and persistent nuclear foci in *ATAD5*-deficient but not in wild type DT40 cells (the cells labelled with the *white asterisk* are enlarged in the insets), while poly(ADP)ribose (PAR) polymer signals were not significantly changed. Data are the mean (± SEM) of three independent experiments. Statistical analysis (two-tailed Student's *t* test) is indicated (*P-value < 0.05).

As in the yeast model system, Dox-induced depletion of ATAD5 in the A2780 clones 1–6 and 15-3 caused a slight increase in the percentage of S phase cells in the unsynchronized cell population as compared to ATAD5-expressing cells (Supplementary Figure S3B), which implied that the cells might have problems during replication. We were therefore interested to learn, whether the genomic instability and delayed progression through S phase in ATAD5-deficient cells might be linked to defective maturation of OFs. As recent evidence demonstrated extensive accumulation of poly(ADP)ribose chains (PARs) at unligated OFs (45), we examined PAR accumulation in PCNA-positive wild type and *ATAD5^−^^/^^−^* S phase DT40 cells, in the presence of PDD 0017273 (Tocris Bioscience), an inhibitor of poly(ADP)ribose glycohydrolase (PARGi). As shown in the inset of Figure 4, the ATAD5-deficient S/G2 phase cells had enlarged chromatin-bound PCNA foci compared to wild type cells as reported previously (30), but PAR accumulation was not significantly changed upon PARG inhibition (Figure 4). This shows that, like Elg1 deletion in yeast (11), ATAD5 deficiency does not appear to affect maturation of OFs in our experimental setting.

### Olaparib sensitivity of ATAD5-deficient cells is affected by oxidative damage processing

**Figure 5.**
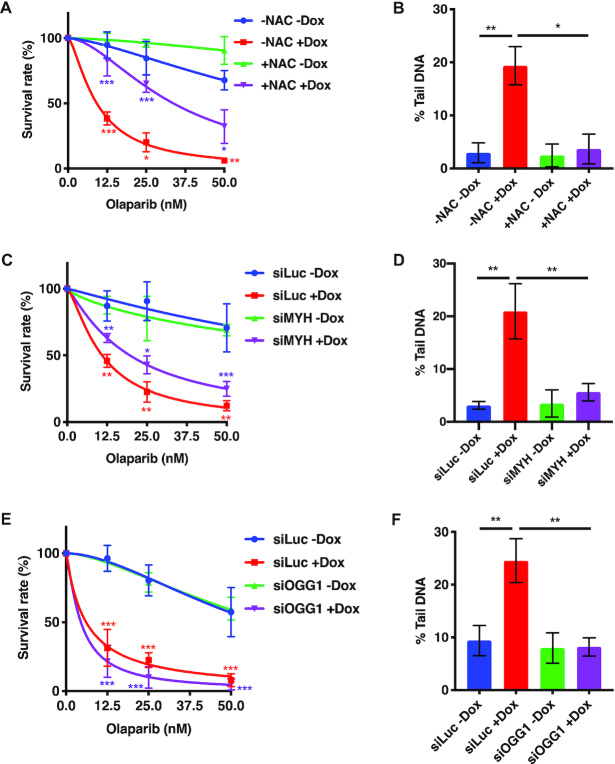
The synthetic lethality of PARP inhibition and ATAD5 deficiency is attenuated by the antioxidant *N*-acetylcysteine, or by depletion of MYH or OGG1 glycosylases. (**A**) Hypersensitivity of ATAD5-depleted human ovarian carcinoma cell line A2780 to olaparib as measured by clonogenic assays was partially rescued by a pre-treatment with the antioxidant *N*-acetylcysteine (NAC). (**B**) NAC treatment attenuated the genotoxicity of ATAD5-depleted cells, as measured by alkaline comet assays. (**C**) MYH knock-down in the A2780 clone 1–6 with siRNA partially rescued the sensitivity of Dox-treated cells to olaparib as assayed by clonogenic assays. (**D**) Accumulation of DNA breaks in Dox-treated A2780 cells was reduced by siRNA-mediated MYH depletion, as estimated by alkaline comet assays. (**E**) OGG1 knock-down in the A2780 clone 1–6 with siRNA failed to rescue the sensitivity of Dox-treated cells to olaparib as assayed by clonogenic assays. (See text for discussion). (**F**) Accumulation of DNA breaks in Dox-treated A2780 cells was reduced by siRNA-mediated OGG1 depletion, as estimated by alkaline comet assays. The results are means of at least three independent experiments, each carried out in triplicate ± SD. Asterisks indicate levels of statistical significance calculated by two-tailed Student's *t* test (*P*-value < 0.05 *, < 0.01 **, < 0.001 ***).

We recently demonstrated (50) that PARP toxicity in BRCA1-depleted or -deficient cells can be attenuated by reducing the amount of spontaneous oxidative DNA damage, the processing of which by BER gives rise to transient SSBs and thus to genomic instability in HR-deficient cells that becomes cytotoxic upon inhibition of PARPs. We argued that if ATAD5-depleted cells were indeed defective in HR, then their sensitivity to PARPis should be similarly rescued by ROS scavengers or inhibition of oxidative DNA damage processing by BER. In order to find out whether this was the case, we treated the cells with the reactive oxygen species (ROS) scavenger *N*-acetylcysteine (NAC) prior to exposing them to the PARPi. The ROS scavenger significantly reduced olaparib toxicity in A2780 cells grown in Dox (Figure 5A and Supplementary Figure S4A) and also the genomic instability in ATAD5-depleted cells, as measured by alkaline comet assays (Figure 5B and Supplementary Figure S4B).

BER of oxidative damage is initiated primarily by two enzymes: oxoguanine DNA glycosylase (OGG1) and MutY homolog DNA glycosylase (MYH, also known as MUTYH). OGG1 is a glycosylase/lyase that removes 8-oxoguanine (G^o^) from G^o^/C pairs arising by oxidation of double-stranded DNA and simultaneously cleaves the resulting abasic site at its 3′ side by β-elimination. AP-endonuclease then cleaves the 5′side to remove the baseless sugar-phosphate to generate a single nucleotide gap. Polymerase-β subsequently inserts a dGMP residue to restore a G/C pair. MutY homolog DNA glycosylase (MYH, also known as MUTYH) removes adenines from A/G^o^ mispairs arising by incorporation of an A opposite G^o^ in the template strand during replication. BER then removes the abasic site and inserts a dC residue opposite the oxidised guanine to generate a G^o^/C pair, which is subsequently processed to G/C by OGG1-initiated BER as described above [see ref (51) for review]. We wanted to test whether, as in the case of BRCA1-depleted cells (50), olaparib sensitivity of the ATAD5-deficient cells would be attenuated by depletion of these DNA glycosylases. The rationale being that if Go/A and/or Go/C processing is inhibited, the number of SSBs and thus PARP binding sites will be reduced, which should reduce the likelihood of the unrepaired breaks colliding with replication forks, giving thus rise to cytotoxic DSBs. Knock-down of MYH did indeed bring about a small but reproducible rescue of olaparib toxicity (Figure 5C and Supplementary Figure S4C) and an attenuation of genomic instability (Figure 5D and Supplementary Figure S4D) in A2780 cells cultured in Dox. As shown in Figure 5E, no rescue of olaparib toxicity was apparent in the clonogenic assays upon OGG1 knock-down in ATAD5-depleted cells, although genomic instability in these cells was attenuated to a similar extent as seen upon MYH downregulation (Figure 5F). We have currently no mechanistic explanation for this anomalous result. OGG1 has been reported to play an important role in mitochondrial metabolism (52) and chemical OGG1 inhibitors appear to arrest the growth of several transformed human cell lines (T. Helleday, personal communication), possibly also because of inhibition of the mitochondrial OGG1 isoform. This agrees with our observation that the growth of our cell clones was retarded upon OGG1 knock down. However, this growth defect was substantially augmented when ATAD5 was also depleted: when equal cell numbers were seeded, ∼80% fewer colonies of OGG1 siRNA-treated cells appeared, as compared to the control, siLuc-treated cells. It is therefore conceivable that the attenuating effect of OGG1 knock-down on olaparib genotoxicity seen in the comet assays of ATAD5-deficient cells (Figure 5F) is not apparent in the clonogenic assays, most likely because the defective mitochondrial fuction caused by the OGG1 defect, coupled to the slow growth of ATAD5-deficient cells (Supplementary Figure S1A), affects their metabolism much more severely over the two-week time course of the clonogenic assays as compared to the brief incubation period used in the comet assays.

In summary, ATAD5 deficiency appears to cause genomic instability through several distinct mechanisms, most of which appear to be connected to the trapping of the polymerase processivity factor PCNA on DNA. This is likely to prevent DSB resection during the initial phase of HR and thus also the loading of RPA and RAD51, as seen in the decrease of RPA foci in CPT-treated cells (Figure 1E). Indeed, most recent evidence implicates ATAD5 in the loading of RAD51 on ssDNA during HR (53). The inhibition of HR by trapped PCNA would channel the DSBs arising at replication forks through collision with SSBs into NHEJ (seen as an increase in 53BP1 foci in the CPT-treated ATAD5-depleted cells in Figure 1F), which would result in genomic instability. Our data implicate also the processing of oxidative DNA damage by MYH among these pathways. The link between oxidative damage repair, ATAD5 deficiency and PARPi sensitivity is likely to be of substantial relevance; given that *ATAD5* haploinsufficiency predisposes to cancer, it is likely that mutations at this locus, which have to date been identified in endometrial and ovarian cancer (26,27), may be found in additional tumour types. These are likely to be responsive to PARPi therapy, but our findings suggest that hypoxic tumour regions might be resistant to these drugs. However, given that ATAD5-deficient cells appear to be sensitive to CPT and MMC (and likely also to other DNA-modifying drugs acting as replication barriers, such as platinum compounds), combination therapy with both these classes of cancer chemotherapeutics may represent a viable approach to the treatment of tumours carrying *ATAD5* mutations.

## Supplementary Material

gkaa255_Supplemental_Files

## Notes

Present address: Marie-Christine Weller, University Hospital Zurich, Department of Oncology and Hematology, Rämistrasse 100, 8091 Zurich, Switzerland.

Present address: Tetsuya Shiota, Department of Surgery, Kobe City Nishi-Kobe Medical Center, Nishi Kojidai 5-7-1, Kobe City Hyogo, Japan.
