# Supplementary material for: ATAD5 deficiency alters DNA damage metabolism and sensitizes cells to PARP inhibition
Source: Nucleic Acids Res. 2020 Apr 16;48(9):4928–39. doi: 10.1093/nar/gkaa255 (PMC7229844; doi:10.1093/nar/gkaa255)
Supplement: gkaa255_Supplemental_Files [file gkaa255_supplemental_files.zip › Giovannini NAR2-Rev SI.pdf]

SUPPLEMENTARY FIGURE LEGENDS

Figure S1: Characterisation of the human ovarian carcinoma A2780 cells inducibly-expressing ATAD5 shRNA. A, A2780 cells depleted of ATAD5 (+Dox) proliferate more slowly than ATAD5-expressing cells (-Dox). **B**, Representative Western blot of extracts of A2780 clone 1-6 after 4-day exposure to doxycycline (+Dox) or DMSO (-Dox) shows efficient depletion of ATAD5. Lamin B1 was used as the loading control. **C**, Representative image of a clonogenic assay demonstrating the inhibition of colony-forming ability of the MMS-treated ATAD5-depleted A2780 clone 1-6 cells. The MMS concentrations used are shown above the images. **D**, ATAD5-depleted cells (+Dox) display fewer and less intense RPA foci upon treatment with CPT than cells expressing ATAD5 (-Dox). For quantification, see Fig. 1E. **E**, ATAD5-depleted cells (+Dox) display more intense 53BP1 foci upon treatment with CPT than cells expressing ATAD5 (-Dox). For quantification, see Fig. 1F.

Figure S2: ATAD5-depleted cells are hypersensitive to olaparib. A, Representative image of a clonogenic assay demonstrating the inhibition of colony-forming ability of the ATAD5-depleted A2780 clone 1-6 cells treated with the indicated concentrations of olaparib.
B, Representative Western blot showing efficiency of ATAD5 knock-down in U2OS cells treated with ATAD5 siRNA compared to control cells treated with siLuc. Lamin B1 was used as loading control. **C**, Schematic representation of the chicken *ATAD5* locus (top line), and of the two gene disruption constructs carrying the puromycin (middle line) and histidinol D (bottom line) selection cassettes. The binding site of the Southern blot probe is also shown, together with the *Hin*dIII site positions and expected fragment lengths used in the identification of the knock-out clones by Southern blot analysis (**D**). **E**, RT-PCR analysis of the DT40 WT, ATAD5-/+ and ATAD5-/- clones used in this study. As shown, no ATAD5 mRNA was detected in the knock-out clone. **F**, Similarly to human ATAD5-depleted cells, the DT40 *ATAD5*$^{-/-}$ cells also accumulate PCNA in their chromatin, as shown in the Western blot of their nuclear extracts. Lamin B1 was used as the loading control.

Figure S3: Accumulation of PCNA in the chromatin and a slight accumulation in S phase of cells depleted of ATAD5.
A, A representative immunoprecipitation showing accumulation of PCNA in the chromatin extracts of A2780 cells depleted of ATAD5 (+Dox, lanes 3 and 4) and of PARP1 in

chromatin extracts of ATAD5-depleted cells (+Dox) treated with olaparib (lane 4), as compared with cell extracts of olaparib-treated ATAD5-expressing (-Dox) cells (lane 2). IgG light chain was used as the loading control. **B,** Accumulation of PCNA in the chromatin of U2OS cells transfected with siRNA against luciferase (siLuc, control), ATAD5, PARP1 or both ATAD5 and PARP1, as indicated. A representative example of Western blots of at least three independent experiments is shown. Lamin B1 was used as the loading control. **C**, Depletion of ATAD5 in A2780 clones 1-6 and 15-3 by induction of ATAD5 shRNA with doxycycline (+Dox) caused a slight increase in the proportion of S-phase cells in this unsynchronised cell population.

Figure S4: Treatment of ATAD5-depleted cells with NAC or MYH siRNA attenuates their sensitivity to olaparib. **A**, Representative image of a clonogenic assay demonstrating the rescue of colony-forming ability of the ATAD5-depleted A2780 clone 1-6 cells treated with the indicated concentrations of olaparib following a pre-treatment with the free radical scavenger *N*-acetylcysteine (NAC). **B**, Representative alkaline comets of ATAD5-expressing (-Dox) or depleted (+Dox) cells treated or not with NAC. **C**, Representative image of a clonogenic assay demonstrating the rescue of colony-forming ability of the ATAD5-depleted A2780 clone 1-6 cells treated with the indicated concentrations of olaparib following a pre-treatment with MYH siRNA. **D**, Representative alkaline comets of ATAD5-expressing (-Dox) or depleted (+Dox) cells treated with siRNA against MYH or with siLuc (control). Asterisks indicate levels of statistical significance calculated by two-tailed Student's *t* test (p-value < 0.05 *, < 0.01 **, < 0.001 ***).
