# Supplementary figures and images for: ATAD5 deficiency alters DNA damage metabolism and sensitizes cells to PARP inhibition
Source: Nucleic Acids Res. 2020 Apr 16;48(9):4928–39. doi: 10.1093/nar/gkaa255 (PMC7229844; doi:10.1093/nar/gkaa255)

Fig. S1

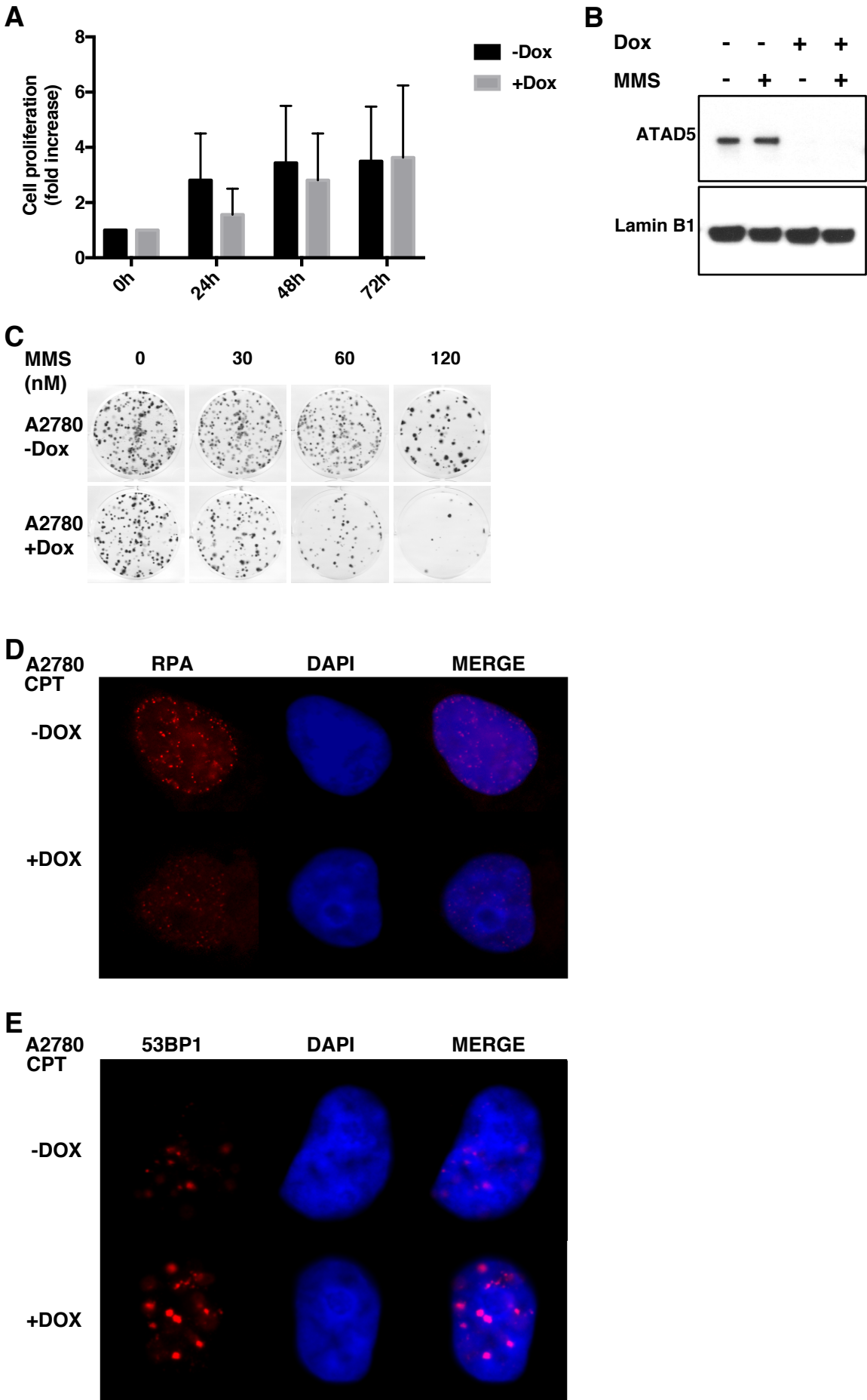

Fig. S2

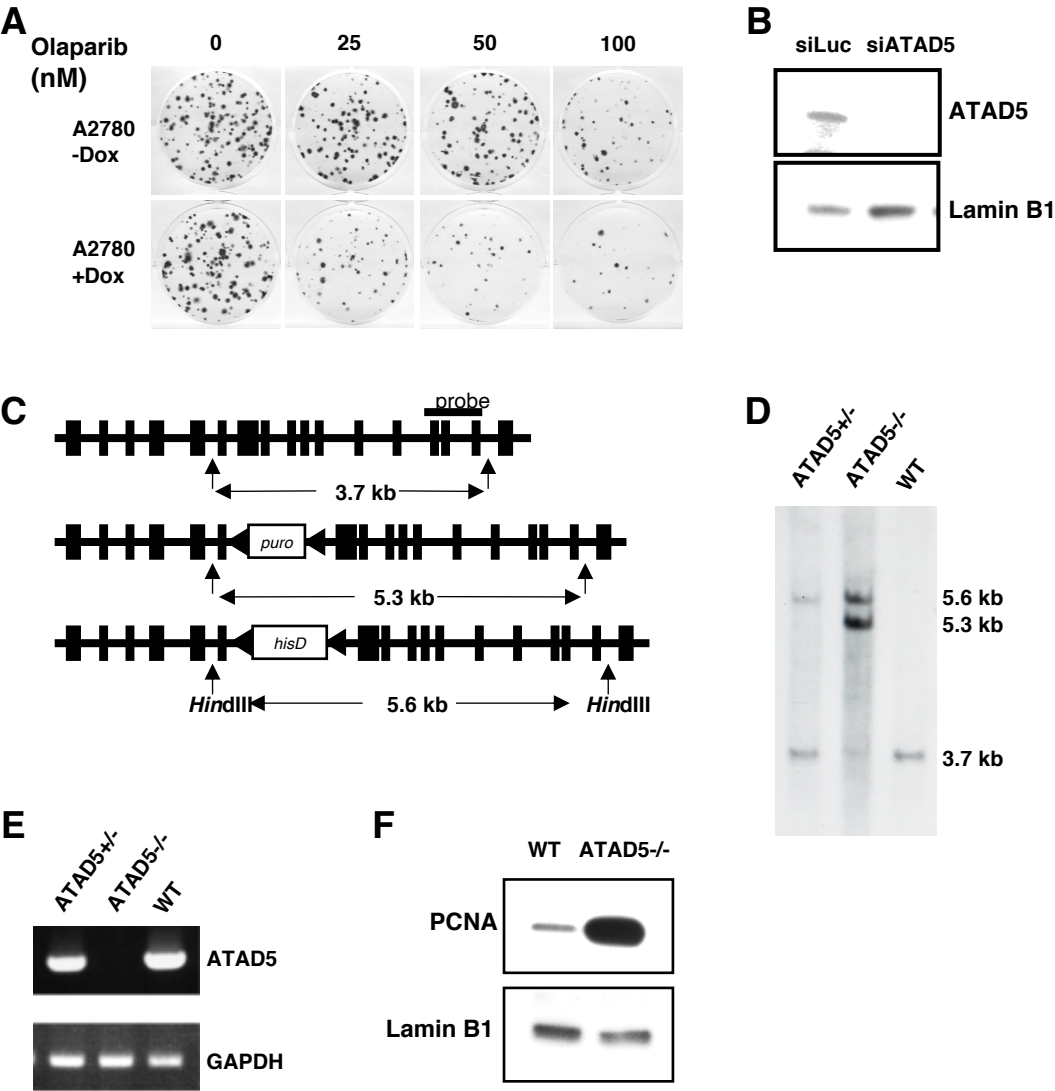

Fig. S3

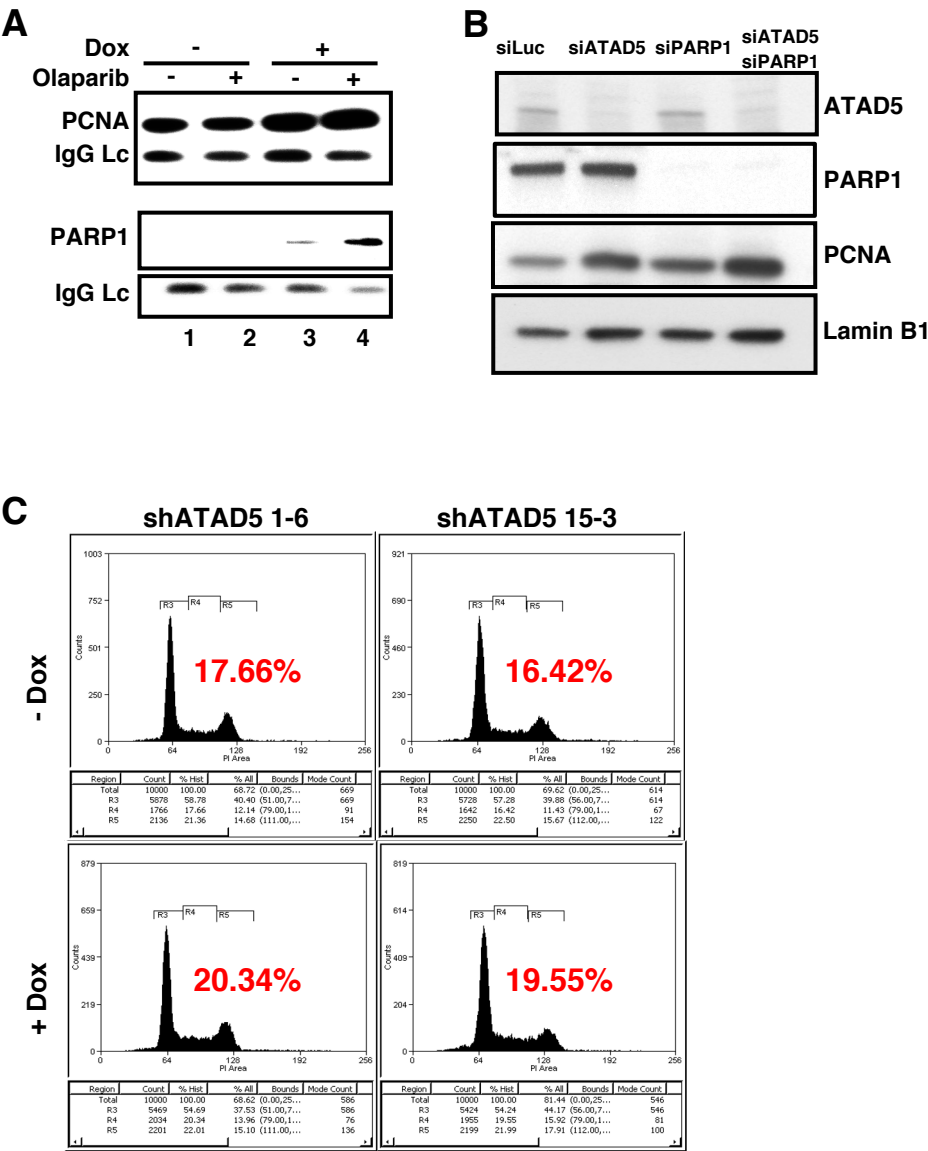

Fig. S4

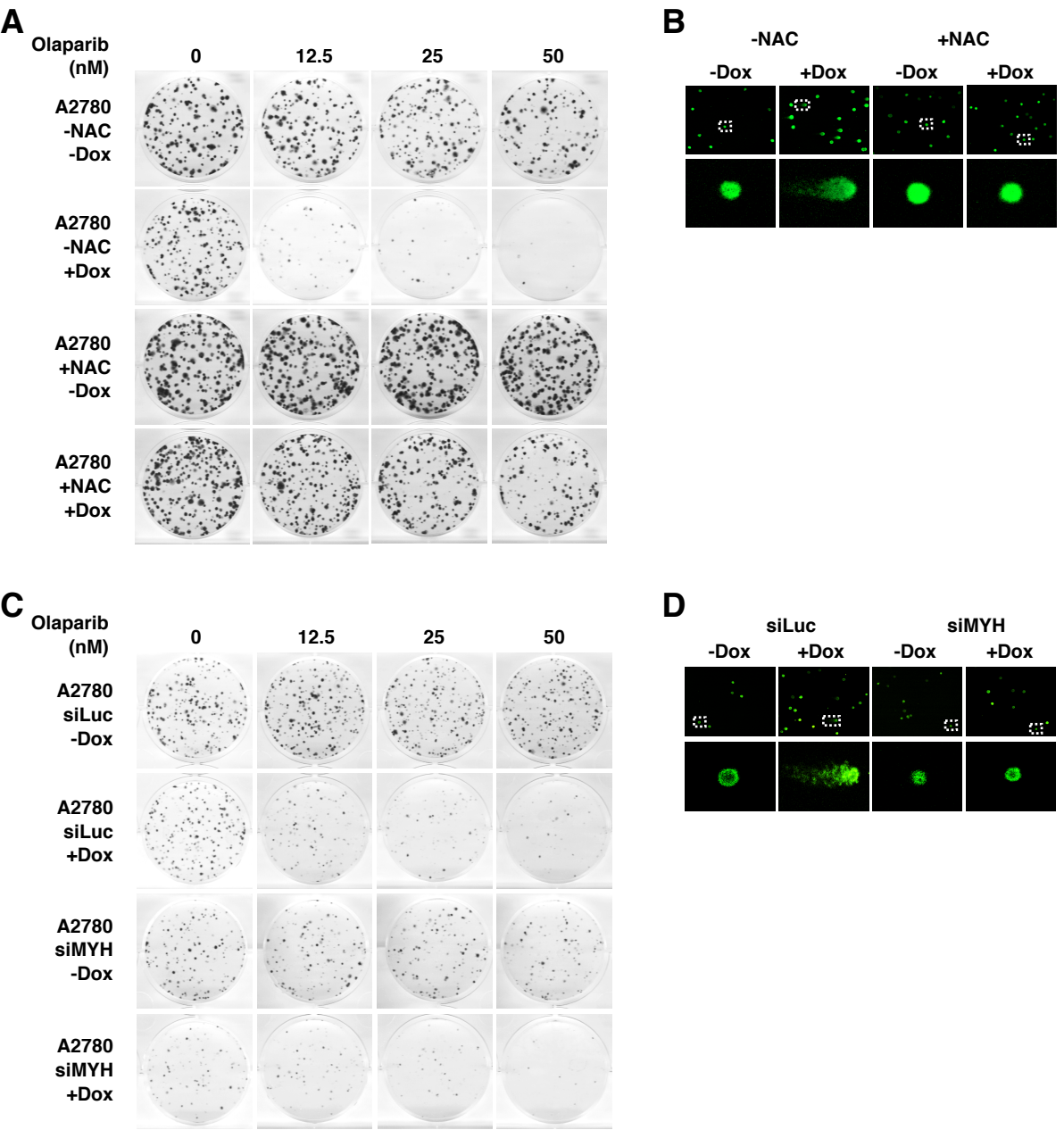

Supplement: gkaa255_Supplemental_Files [file gkaa255_supplemental_files.zip › Giovannini SFigsNAR2-Rev.pdf]
